# Non-invasive Ventilation in Diaphragmatic Paralysis Associated With Neonatal Brachial Plexus Palsy

**DOI:** 10.7759/cureus.92374

**Published:** 2025-09-15

**Authors:** Inês Ferreira Costa, Ana Freitas, Vanessa Silva, Ana Ramos, Lurdes Morais

**Affiliations:** 1 Pediatrics Department, Centro Materno-Infantil do Norte Albino Aroso, Centro Hospitalar Universitário de Santo António, Porto, PRT; 2 Neonatal Intensive Care Unit, Neonatology Department, Centro Materno-Infantil do Norte Albino Aroso, Centro Hospitalar Universitário de Santo António, Porto, PRT; 3 Pediatric Pulmonology Unit, Pediatrics Department, Centro Materno-Infantil do Norte Albino Aroso, Centro Hospitalar Universitário de Santo António, Porto, PRT

**Keywords:** brachial plexus injury (bpi), diaphragmatic paralysis, neonatal respiratory distress, non-invasive ventilation, shoulder dystocia

## Abstract

Obstetric brachial plexus injury (OBPI) is an uncommon yet well-recognized complication of childbirth, occasionally accompanied by phrenic nerve involvement, causing diaphragmatic paralysis. Management of these cases often requires advanced respiratory support, with limited reports describing the use of early non-invasive ventilation (NIV) as first-line therapy. We report a full-term female neonate, delivered by vacuum-assisted birth complicated by shoulder dystocia, who developed right upper limb flaccid paralysis, Horner syndrome, and respiratory distress at birth. Imaging confirmed right hemidiaphragm elevation and brachial plexus root avulsion (C7-T1). She was managed exclusively with early NIV, starting with nasal continuous positive airway pressure and subsequently bilevel ventilation, avoiding invasive support. Enteral feeding and physiotherapy began promptly. By 10 months, NIV was required only during sleep; brachial plexus repair and diaphragmatic plication were performed, with complete weaning by age 2. At 4 years, she shows mild thoracic asymmetry, limited wrist mobility, and a mild speech disorder, with otherwise normal growth and age-appropriate development. This case supports early NIV as a first-line option in neonatal diaphragmatic paralysis secondary to OBPI, challenging conventional reliance on invasive ventilation and promoting a less invasive, outcome-focused approach.

## Introduction

The brachial plexus, formed by the cervical nerves C5 to C8 and the first thoracic nerve (T1), innervates the upper limb. Additionally, the phrenic nerve, which arises primarily from C3-C5, provides motor innervation to the diaphragm and plays a major role in respiratory function [1,2]. Obstetric brachial plexus injury (OBPI) is a relatively common birth trauma, occurring in approximately 0.4% of live vaginal deliveries, most frequently associated with shoulder dystocia [1-3]. OBPI typically presents at birth as a flaccid, unilateral paralysis of the affected upper limb. The most common form, Erb’s palsy, involves injury to the upper roots (C5-C6), whereas the less frequent Klumpke’s palsy affects the lower plexus (C8-T1) and may be associated with Horner syndrome [1,4]. In severe cases, involvement of the phrenic nerve can result in diaphragmatic paralysis, potentially leading to significant respiratory distress [5]. Management of these infants is often complex and requires a multidisciplinary approach, involving neonatologists, pediatric neurologists and pulmonologists, pediatric surgeons, and physiatrists. While invasive ventilation is commonly required in cases of diaphragmatic paralysis, reports of successful NIV are rare [6,7,8]. We report a rare case of a neonate with OBPI and associated diaphragmatic paralysis successfully managed with early NIV, avoiding the need for intubation and invasive respiratory support.

## Case presentation

**Figure 1:**
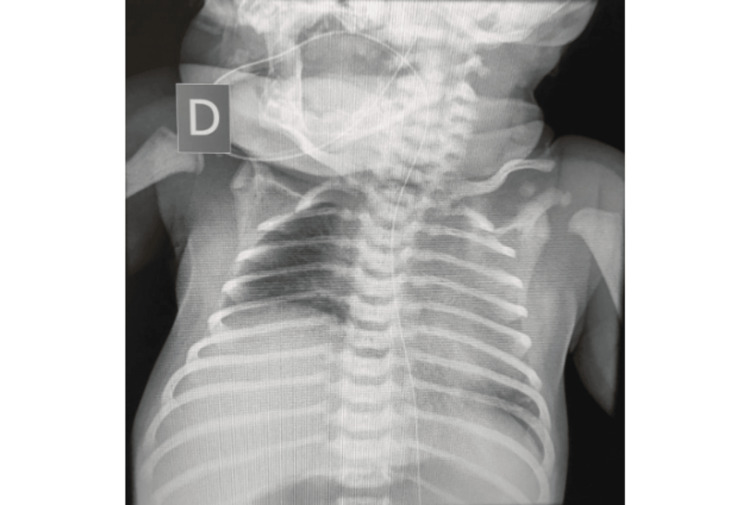
Chest X-ray showing marked elevation of the right hemidiaphragm, consistent with diaphragmatic paralysis.

A full-term female neonate, with an unremarkable prenatal history, was delivered via vacuum-assisted vaginal birth complicated by shoulder dystocia. Her birth weight was 3600g (between the 50th and 85th percentiles for gestational age, according to the Fenton Growth Chart). Her Apgar score was 6 at the 1st minute and 9 at the 5th minute. Neonatal resuscitation with positive pressure ventilation was required due to generalized hypotonia and ineffective respiratory movements. She presented with persistent respiratory distress characterized by tachypnea, subcostal retractions, and oxygen desaturation, requiring ongoing ventilatory support. Further physical examination revealed flaccid paralysis of the right upper limb, thoracic asymmetry, right-sided ptosis, and anisocoria. Due to respiratory and feeding difficulties, as well as worsening of the upper limb function, the patient was admitted to the neonatal intensive care unit (NICU) for ventilatory support. A chest X-ray showed elevation of the right hemidiaphragm (Figure 1), and MRI showed diaphragmatic elevation and root avulsion of the brachial plexus from C7 to T1. The respiratory compromise was attributed to right-sided diaphragmatic paralysis secondary to phrenic nerve injury.

**Figure 2:**
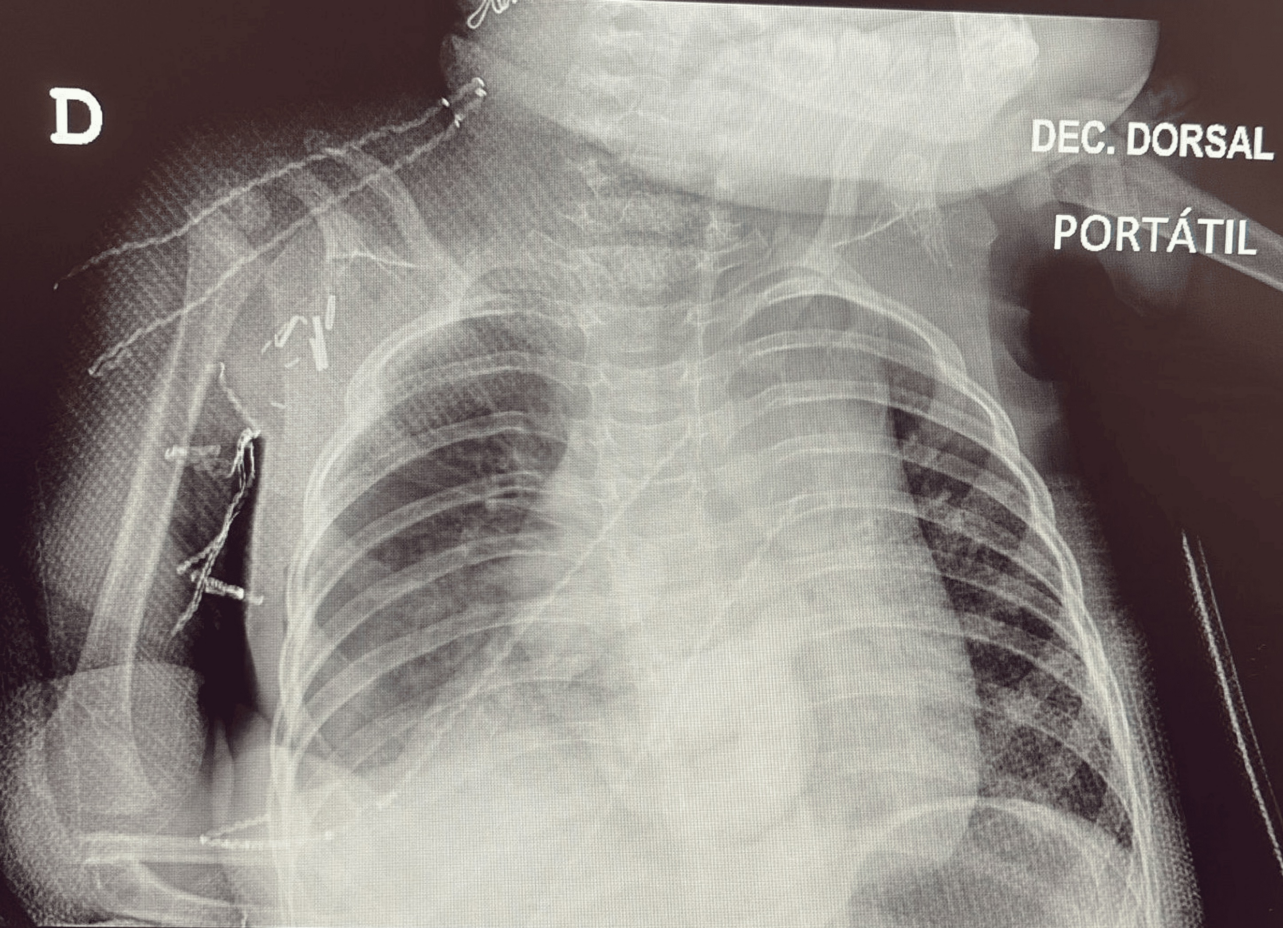
Postoperative image of the patient following brachial plexus repair and diaphragmatic plication.

She was initially managed with nasal continuous positive airway pressure (nCPAP), followed by bilevel NIV (BiPAP) with a portable ventilator Trilogy® 100 (Philips Respironics, USA), a device also suitable for home use (inspiratory positive airway pressure (IPAP) 12 cmH₂O, expiratory positive airway pressure (EPAP) 6 cmH₂O, respiratory rate 50 breaths/min, and inspiratory time 0.5 seconds). A full-face mask was initially used to optimize ventilation, followed by a nasal interface as her respiratory status improved. Alongside respiratory support, early enteral feeding via nasogastric tube and physical therapy were promptly started. Over time, her respiratory distress subsided, and she was discharged home at 3 months of age under NIV with the same Trilogy® 100 device in domiciliary mode. Despite an early multidisciplinary approach, she continued to experience oropharyngeal dysphagia with feeding difficulties, upper limb limitations, and persistent ventilatory support needs. She was prompted for referral for surgical evaluation and regular physical therapy. At 10 months, she only required ventilatory support during sleep periods, and nasogastric feeding was limited to liquids. She underwent surgical brachial plexus repair and diaphragmatic plication after some initial clinical improvement (Figure 2).

Subsequent follow-up revealed steady clinical improvement, with progressive weaning from NIV support, which was successfully discontinued by 2 years of age. Neither major complications related to NIV nor respiratory infections were reported. She continues to undergo regular physical, occupational, and speech therapies. At 4 years of age, she presents with only mild thoracic asymmetry and limited wrist extension/flexion. Her height and weight are appropriate for her age, and despite motor impairments related to brachial injury and a mild speech disorder, her overall developmental progress remains satisfactory.

## Discussion

The occurrence of perinatal diaphragmatic paralysis associated with brachial plexus palsy is rare but carries significant morbidity and mortality. The estimated mortality rate, often linked to pneumonia or extensive atelectasis, ranges from 10 to 15% [9]. Recognized risk factors for phrenic nerve injury include breech presentation, shoulder dystocia, and the use of forceps or vacuum extraction [10]. Our case aligns with existing literature, as the right hemidiaphragm is predominantly affected in 70-80% of cases [8, 10].

Currently, there are no established guidelines for the management of these patients, and initial treatment commonly involves mechanical ventilation and nasogastric tube feeding [4]. Surgical intervention is frequently required due to severe respiratory distress and failure to thrive, with optimal timing generally recommended within the first 9 months of life, though this remains controversial [4, 11, 12].

In addition to diaphragmatic plication, brachial plexus reconstruction is essential to prevent long-term upper limb functional deficits and support diaphragmatic recovery [11]. Literature suggests that a conservative approach involving prolonged conventional mechanical ventilation may improve respiratory function and potentially obviate the need for surgery [13].

NIV is typically introduced following a period of invasive mechanical ventilation. However, fewer than 10 cases in the literature report NIV as the primary and effective modality of ventilatory support. By providing continuous positive airway pressure and sufficient transpulmonary pressure to prevent alveolar collapse, NIV maintains lung volume and facilitates progressive diaphragmatic function recovery. Moreover, by allowing spontaneous breathing and preserving physiological respiratory mechanics, NIV should be considered before invasive ventilation strategies, thereby minimizing associated complications [8,14].

In our patient, NIV was initiated as the first-line ventilatory support, resulting in significant respiratory improvement and partial diaphragmatic recovery without requiring invasive ventilation. By 9 months, NIV was necessary only during sleep, and following surgical intervention, ventilatory support was completely discontinued, thereby highlighting the effectiveness and therapeutic potential of this ventilatory modality.

## Conclusions

This case underscores the advantages of NIV as a less invasive approach with clear clinical benefits. Beyond promoting a more physiological respiratory course for the newborn, NIV may also lessen the psychological burden on families and contribute to a better quality of life. Early NIV support should be considered as a first-line strategy in neonates with diaphragmatic paralysis secondary to OBPI, as it may reduce the need for invasive ventilation and its associated complications.
